# Human sperm rotate with a conserved direction during free swimming in four dimensions

**DOI:** 10.1242/jcs.261306

**Published:** 2023-11-29

**Authors:** Gabriel Corkidi, Fernando Montoya, Ana L. González-Cota, Paul Hernández-Herrera, Neil C. Bruce, Hermes Bloomfield-Gadêlha, Alberto Darszon

**Affiliations:** ^1^ Laboratorio de Imágenes y Visión por Computadora, Departamento de Ingeniería Celular y Biocatálisis, Instituto de Biotecnología, Universidad Nacional Autónoma de México, Cuernavaca 62210, México; ^2^ Departamento de Genética del Desarrollo y Fisiología Molecular and Instituto de Biotecnología, Universidad Nacional Autónoma de México, Cuernavaca 62210, México; ^3^ Laboratorio Nacional de Microscopía Avanzada, Instituto de Biotecnología, Universidad Nacional Autónoma de México, Cuernavaca 62210, México; ^4^ Instituto de Ciencias Aplicadas y Tecnología, Universidad Nacional Autónoma de México, Circuito Exterior S/N, Ciudad Universitaria, 04510 Ciudad de México, México; ^5^ School of Engineering Mathematics and Technology & Bristol Robotics Laboratory, University of Bristol, Bristol BS8 1TW, UK

**Keywords:** 3D+t, Sperm motility, Head rotation, Multi-plane imaging, Spherical aberration

## Abstract

Head rotation in human spermatozoa is essential for different swimming modes and fertilisation, as it links the molecular workings of the flagellar beat with sperm motion in three-dimensional (3D) space over time. Determining the direction of head rotation has been hindered by the symmetry and translucent nature of the sperm head, and by the fast 3D motion driven by the helical flagellar beat. Analysis has been mostly restricted to two-dimensional (2D) single focal plane image analysis, which enables tracking of head centre position but not tracking of head rotation. Despite the conserved helical beating of the human sperm flagellum, human sperm head rotation has been reported to be uni- or bi-directional, and even to intermittently change direction in a given cell. Here, we directly measure the head rotation of freely swimming human sperm using multi-plane 4D (3D+t) microscopy and show that: (1) 2D microscopy is unable to distinguish head rotation direction in human spermatozoa; (2) head rotation direction in non-capacitating and capacitating solutions, for both aqueous and viscous media, is counterclockwise (CCW), as seen from head to tail, in all rotating spermatozoa, regardless of the experimental conditions; and (3) head rotation is suppressed in 36% of spermatozoa swimming in non-capacitating viscous medium, although CCW rotation is recovered after incubation in capacitating conditions within the same viscous medium, possibly unveiling an unexplored aspect of the essential need of capacitation for fertilisation. Our observations show that the CCW head rotation in human sperm is conserved. It constitutes a robust and persistent helical driving mechanism that influences sperm navigation in 3D space over time, and thus is of critical importance in cell motility, propulsion of flagellated microorganisms, sperm motility assessments, human reproduction research, and self-organisation of flagellar beating patterns and swimming in 3D space.

## Teaser

**Summary:** We utilise the spherical aberration of the objective lens in bright-field imaging to reveal that the human spermatozoon head rotates in a counterclockwise direction, as viewed from head to tail.

## INTRODUCTION

Mammalian spermatozoa invariably rotate as they freely swim through a fluid. Coordinated helical motion of its whip-like flagellum causes the spermatozoon to ‘corkscrew’ into the fluid, leading to rotation of the spermatozoon head around its longitudinal axis during locomotion (David et al., 1981; Denehy et al., 1975; Ishijima et al., 1992; Linnet, 1979; 1981; Phillips, 1972; Rikmenspoel, 1965; Woolley, 1977; Woolley and Osborn, 1984). Sperm head rotation is a direct manifestation of the cyclic molecular motor activity shaping the flagellum into a helical beat in three-dimensional (3D) space (Woolley, 1977; Woolley and Osborn, 1984): a right-handed helical flagellar beat causes the sperm head to rotate clockwise (CW), whereas a sperm with a left-handed helical flagellar beat rotates in the opposite direction when seen from head to tail. Sperm rotation is suppressed in a subset of spermatozoa if the flagellar waveform is purely planar [i.e. two-dimensional (2D)], as happens when human sperm penetrate viscous medium (Smith et al., 2009a). Head spinning is thus a fundamental feature linking the molecular workings of the flagellar beat with sperm motion in 3D space (Zaferani et al., 2021a). The symmetry state of the flagellar beat cannot be fully evaluated without knowledge of head rotation and its direction in 3D space. The modulation of symmetry is critical for sperm navigation in three dimensions, for instance in chemotaxis and hyperactivation (Chang and Suarez, 2010; Ren and Bloomfield-Gadêlha, 2023 preprint). Head rotation is thus important for successful fertilisation and is related to a complex cascade of transmembrane ion channel transport that coordinates this mode of motion (Miller et al., 2018; Zhao et al., 2022).

In the past decades, numerous researchers have endeavoured to ascertain the direction of spermatozoon head rotation through a range of methodologies. This pursuit is driven by the fundamental need for data on both the sense and the rate of rotation, as these parameters are essential for establishing the 3D geometry of flagellar motion (Ishijima et al., 1992). However, to date, there has been no consensus. Studies of human sperm have reported unidirectional rotation (Linnet, 1979; 1981; Smith et al., 2009b; Phillips, 1983; Woolley, 1977), bi-directional rotation (Ishijima et al., 1992; Dardikman et al., 2020; Drake, 1974) and even intermittent changes in the direction of rotation (Bukatin et al., 2015). There is no agreement among earlier studies as to the reported direction of rotation (Woolley, 1977; Bishop, 1958; Drake, 1974; Yeung and Woolley, 1984; Woolley, 1979; Blokhuis, 1961; Daloglu et al., 2018). Such reported variety in rotation direction appears to contradict observations of a conserved helical beating of the human sperm flagellum (Bukatin et al., 2015; Ishijima et al., 1992; Linnet, 1979; 1981; Powar et al., 2022; Zhao et al., 2022) and conserved chirality of structural components in mammalian sperm flagella (Fawcett, 1975; Leung et al., 2021).

**Fig. 1.**
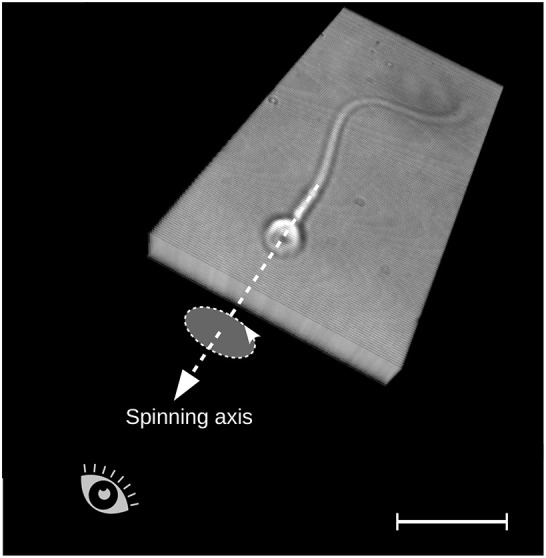
**The human sperm head is translucent and has an axial symmetric morphology around its rotating axis.** Image depicts a 3D multi-plane stack capturing a free-swimming spermatozoon, presented as a volume rendering, while propelling forward and causing its head to rotate around its longitudinal axis during locomotion. The axis of rotation is marked by the dashed line. The reference of observation in this work is from head to tail, as indicated by the eye symbol. Scale bar: 10 µm.

**Fig. 2.**
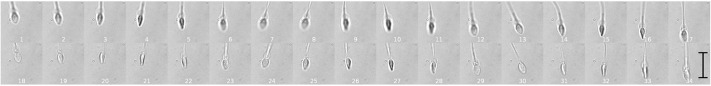
**Contrast of the sperm head is dependent on focal plane.** 2D single-plane image sequences of 17 consecutive timepoints (total time 189 ms) at two different focal planes separated by 4.8 µm (images 1–17, upper focal plane; images 18–34, lower focal plane), containing the information of a complete head turn for a spermatozoon in non-capacitating and low viscosity conditions (see Movie 1). Scale bar: 10 µm.

The human sperm head is translucent and has a planar axial symmetrical morphology around its rotating axis (Fig. 1), similar to a flattened ellipsoid (see also Fig. 6A, images 1 and 6), making it prone to optical illusions that can obscure accurate detection of the directionality of rotation. As such, the sperm head is prone to perception bistability, the spontaneous switching between two or more interpretations of an image under continuous viewing (Devia et al., 2022), which can further disguise the true direction of rotation in translucent objects. This optical illusion causes spinning translucent objects to appear to oscillate back and forth, or spin with a switchable and thus undefined direction (Liu et al., 2012). At a microscale, this difficulty is augmented by the contrast inversion effect due to spherical aberrations in microscope objectives (which are different from other aberrations such as coma or astigmatism, as they are independent of the object position – at the centre or edges – over the whole field of view), which causes a contrast switch depending on the position of the object relative to the plane of focus (Goodman, 2005; https://www.olympus-lifescience.com/en/microscope-resource/primer/java/aberrations/pointspreadaberration/). The switching of the contrast of spinning objects and its subsequent dependence on the focal plane used for imaging have unknown consequences for detection of the direction of spinning (Fig. 2), as we further detail in this paper. Taken together, these challenges indicate that 2D microscopy may not permit accurate measurement of the directionality of sperm head rotation (Muschol et al., 2018). Indirect detection methods either exploit sperm head centre position traces (Ishijima et al., 1992; Yániz et al., 2020; Gallagher et al., 2019) or track a certain point along the flagellum in 3D space (Ishijima et al., 1992; Bukatin et al., 2015; Dardikman et al., 2020). In these approaches it is assumed that the head rotation direction follows a similar rotational movement as the position traces of these counterparts. Indirect detection studies, however, have not yet been validated against direct measurements of head rotation, as no ground truth is available for this, highlighting an urgent gap in the literature. This is, however, critical for understanding sperm swimming, as human sperm precess around the swimming axis, where the sperm head remains firmly attached to the connecting piece that anchors the flagellum (Lindemann and Lesich, 2016), rotating as a whole around its swimming direction. Indeed, recent high-resolution cryo-electron tomography enabled by cryo-focused ion beam milling has revealed novel protofilament-bridging microtubule inner proteins at the asymmetric mammalian sperm centrioles, which might be key to allow progressive swimming in viscous media (Leung et al., 2021; Khanal et al., 2021).

Here, we solve this half-century-old problem by using a high-resolution, multi-plane four-dimensional (4D) method (Corkidi et al., 2008; 2021; Silva-Villalobos et al., 2014; Pimentel et al., 2012; Gadêlha et al., 2020; 2021; Hernández et al., 2022) to directly detect the head rotation direction of human spermatozoa. We analysed and compared hundreds of human spermatozoa in non-capacitating and capacitating solutions of low- and high-viscosity media, mimicking the female reproductive tract. These comparisons were performed to take into account the effects of capacitation, which is a maturational process occurring in the female reproductive tract that regulates sperm swimming and is necessary for fertilisation (reviewed in Gervasi and Visconti, 2016). Considering this, all sperm heads were observed to rotate in a counterclockwise (CCW) direction when viewed from head to tail (Fig. 1). The rotation direction in human spermatozoa is conserved, and even cells in non-capacitating high-viscosity medium that display planar flagellar beating acquire CCW head rotation after capacitation in viscous medium, indicating the presence of a conserved helical driving mechanism powering the human sperm flagellum. These observations could have important implications for our understanding of the internal machinery that drives head-rotating molecular motor-powered flagellar cell motility and its physiology.

## RESULTS

### The direction of head rotation cannot be determined confidently using 2D single-plane imaging

To directly determine the direction of rotation of the head of human spermatozoa we implemented a bright-field microscopy multi-plane 4D detection system (also referred to here as 3D+t microscopy), which is described in detail in the Materials and Methods section. The system takes advantage of the contrast inversion that occurs while the head is rotating. This requires finding the appropriate focal plane in which the sperm head is centred exactly at the focal plane; a challenging task for freely swimming spermatozoa, as the head moves up and down. Determining this precise plane requires the multi-plane 4D detection system. As described below, the direction of head rotation could not be established without ambiguity by performing 2D imaging experiments.

The 2D single-plane image sequences in Fig. 2, with a left-to-right direction, show two different focal planes containing the information of a complete head turn. As can be appreciated, the visual information in these two planes is distinct and complex, with changes in the brightness, contrast switches and head position relative to the focal plane. Establishing the direction of head rotation was not possible using single-plane information alone (see Movie 1). Visual inspection by different observers led to different assessments of rotation direction due to bistability perception (data not shown). These observations using our system indicate that classical 2D single-plane imaging is not suitable to establish confidently the direction of the head rotation.

### Acquisition of 3D bright-field image stacks with contrast inversion for the human sperm head

**Fig. 3.**
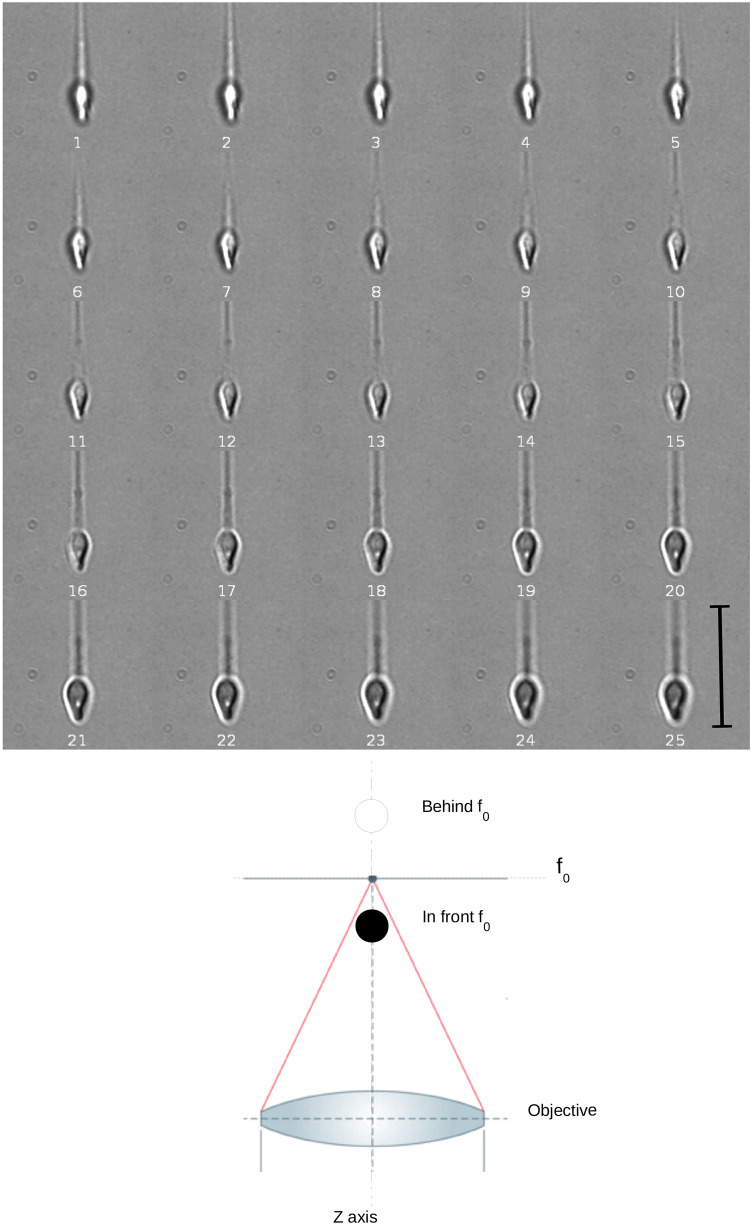
**Acquisition of images at multiple focal planes.** Top: images showing a numbered sequence of 25 consecutive focal planes (out of 50) acquired during a single rising slope of the piezoelectric device used to displace the objective. The images depict a spermatozoon in non-capacitating and low viscosity conditions. Frames (150×150 pixels each) were cropped for visualisation purposes from 640×480-pixel image sequences. Focal planes are separated by 0.4 µm (see Materials and Methods). Bottom: schematic depicting how the spherical aberration of the optical system produces a contrast inversion over translucent objects depending on their position with respect to the focal plane *f*_0_. The object appears dark when placed in front of the focal plane *f*_0_, whereas it appears bright when placed behind the focal plane. Scale bar: 10 µm.

Fig. 3 shows 25 images of consecutive focal planes acquired during a single rising slope of the piezoelectric device used to displace the objective of the inverted microscope used. The experimental setup allowed us to achieve a temporal and spatial resolution of 1/160 s and 0.4 µm, respectively, along the *z* direction. As can been seen in Fig. 3, different head elements and their contrast inversion were revealed as the focal plane moved through the sperm head, with bright pixels inside the head and a dark halo outside the head when the head was behind the focal plane (Fig. 3, image 1), and with dark pixels inside the head and a bright halo outside the head when the head was in front of the focal plane (Fig. 3, image 25). Note particularly how the left narrowest side of the sperm head began to appear as a bright white border (detailed further below) when the image moved further from the objective lens (Fig. 3, images 11–15). Application of this contrast inversion effect to track head rotation directionality is described in the following section.

### Contrast inversion of the sperm head is due to the spherical aberration of the objective lens used for bright-field microscopy

Rayleigh–Sommerfeld back propagation reconstruction is an approximate numerical method using scalar diffraction theory for reconstruction of 3D objects based on the image or the diffraction pattern of the object; it has commonly been used to track the sperm head and flagellum in 3D space over time (Pimentel et al., 2012; Silva-Villalobos et al., 2014; Bukatin et al., 2015). However, this approach is based solely on diffraction and cannot distinguish between defocus below or above the focused object plane (Lee and Grier, 2007). Usually, the contrast inversion of the defocused object is used heuristically to post-process the results of the back propagation method to produce a final reconstructed object (Lee and Grier, 2007). Alternatively, phase information from the reconstructed object can be used to post-process the reconstruction itself (Wilson and Zhang, 2012). It is well known that microscope objectives are extremely well corrected for conjugate planes, which are focused objects and image planes, but this is not true for non-conjugate planes. Aberrations, particularly spherical aberrations, can strongly contribute to image quality for non-conjugate planes (Kidger, 2001; https://www.olympus-lifescience.com/en/microscope-resource/primer/java/aberrations/pointspreadaberration/). Here, we demonstrate that it is the spherical aberration of the objective lens that produces a contrast inversion as a function of the position of the object relative to the focused object plane (Fig. 3).

**Fig. 4.**
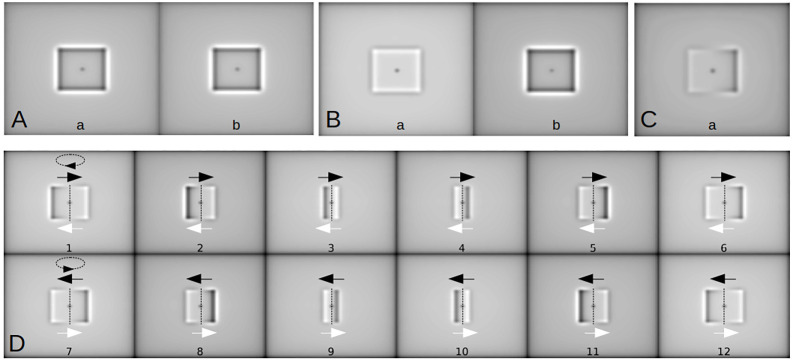
**Simulation of image formation for a rectangular, translucent and weakly scattering phase object and for a spinning one.** (A,B) Simulation of imaging for an object parallel to the objective lens. (A) The simulation on the left (image a) has *A_s_*=0 and *A_d_*=0.3. The simulation on the right (image b) has *A_s_*=0 and *A_d_*=−0.3. (B) The simulation on the left (image a) has *A_s_*=−0.0004 and *A_d_*=0.04. The simulation on the right (image b) has *A_s_*=−0.0004 and *A_d_*=−0.04. (C) Simulation of imaging for an inclined (≈ 5 degrees) planar object with *A_s_*=−0.0004. The centre of the object plate is in the focused object plane of the lens and the right part of the object is closer to the objective lens. (D) Simulation results for a spinning object, showing the direction of movement of the dark edges (black arrows) and bright edges (white arrows) from 2D imaging from a fixed focal plane projection. The direction of rotation is indicated by dashed arrows. Top (images 1–6): a CW rotation viewed from the top of the images. Bottom (images 7–12): a CCW rotation viewed from the top of the images. *A_s_*=−0.004; maximum defocus, *A_d_*=±0.2. _ _

Diffraction effects that appear in the point spread function (PSF) are given by the Fourier transform of the lens aperture function multiplied by the aberration function:
(1)

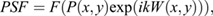


where *F*() is the Fourier transform in the lens aperture plane, (*x*, *y*) is the position in the image plane, *P*(*x*, *y*) is the lens pupil function (almost always taken as a circle), *k*=2*π*/*λ* is the light wavenumber, *i* = √−1 and *W*(*x*, *y*) is the wave aberration function given by Goodman (2005):
(2)

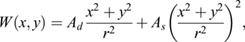


where *A*_*d*_ is the amplitude of the defocus, *A*_*s*_ is the amplitude of the spherical aberration and *r* is the radius of the lens pupil. The first term in Eqn 2 is the defocus contribution, and the second term is the contribution of the spherical aberration. It is important to note that Eqn 2 is independent of the object position, meaning that the contributions of these two aberrations are the same over the whole field of view for a given object and image distance. Another important aspect is the sign of the defocus term. From Goodman (2005), using the Gauss equation for a defocused system we have:
(3)

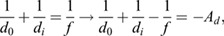


so that *A*_*d*_=0 when the system is focused. Given that *f* and *d*_*i*_ (focal length and the distance from the lens to the image, respectively) are fixed, and that the distance from the object to the lens (*d*_0_) changes when the optical system is defocused, when *d*_0_ increases (the object is further away from the objective lens), the term 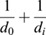 is smaller and *A*_*d*_>0. When *d*_0_ decreases (the object is closer to the objective lens), the term 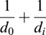 increases and *A*_*d*_<0. We assume that the spherical aberration has the same sign for a defocus below and above the geometrical focal plane. Simulations for a rectangular object are shown in Fig. 4A–C. The diffraction from defocus alone did not give an inversion of contrast between the object positions below and above the focused object plane (Fig. 4A, panels a and b). However, when a small spherical aberration contribution was included, the contrast inversion effect was observed (Fig. 4B, panels a and b). Finally, in the case of an inclined object, with the left part of the object further away from the objective lens and the right part closer to the objective lens, contrast inversion within the object was observed (Fig. 4C). It is important to note that a change in sign of the spherical aberration contribution changed the contrast inversion: with *A*_*s*_>0, an object closer to the objective lens than the focused object plane would be dark and an object further from the objective lens would be bright. The sign of the aberration depends on the specific design of the optical system and so could vary between different laboratories working with different objectives. Our simulations considered a spherical aberration within the range of the optical system used in our experiments.

The contrast switch inside the object is a direct manifestation of the inclination of the object relative to the objective lens (Fig. 4C). Here, we exploited this unique optical effect for the first time to extract the directionality of a spinning object, such as the sperm head, as detailed below. Fig. 4D shows how this contrast switch of the planar projection varies when the inclination of the object increases as the object spins around its long axis. Spinning in a CW direction (Fig. 4D, top) causes the bright part (further away from the objective lens, see white arrows) of the inclined object to always move from right to left and the dark part to move from left to right (dark arrows), whereas CCW spinning (Fig. 4D, bottom) causes the bright region to always move from left to right at the same time that the dark region moves from right to left. Once a half cycle is completed and the flat object is parallel to the objective lens, the bottom part of the object reaches the top, and vice versa, resetting the contrast inversion; any part of the object reaching the top appears as bright, and any part of the object reaching the bottom will appear as dark. In other words, after a half cycle, bright and dark regions abruptly reappear at their starting locations, after reaching the end of the object on the opposite side. For this reason, if an object spins in the same direction, this will be manifested in the image as the bright and dark regions of the object each moving with a persistent direction relative to the orientation of the object (left-to-right or right-to-left). Likewise, if the object spinning direction is reversed, the directions of motion of bright and dark regions will then equally reverse relative to the orientation of the object. The spherical aberration effect causing the contrast switch within the image of the object allows for robust, and yet simple, detection of the spinning direction of objects with axial symmetry, such as the human sperm head (as demonstrated below), that otherwise would not be possible, and this feature is exploited here for the first time.

### The bright region induced by sperm head inclination moves with head rotation

**Fig. 5.**
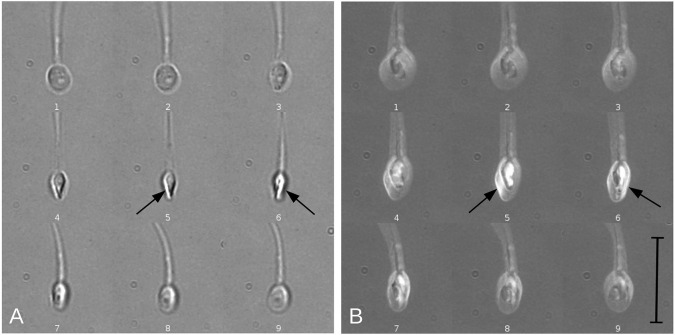
**The contrast inversion optical effect during a half turn of a sperm head.** (A) Numbered sequence of nine consecutive timepoints (0.1 s total time) where the contrast inversion optical effect can clearly be observed for a spermatozoon in non-capacitating and low viscosity conditions. The same focal plane is shown in each image. The narrowest, focally lower, border of the sperm head (behind the focal plane) is enhanced, resulting in a bright border while the sperm head is inclined during head rotation (highlighted with arrows). (B) Numbered sequence of 2D MIPs for the nine consecutive timepoints shown in A, depicting a half turn of a sperm head. The MIP for each timepoint was constructed from the multiple focal planes acquired during a complete rising slope of the piezoelectric device (50 images per projection). The brightest pixels over the *z* axis from 50 images corresponding to one half cycle of the piezoelectric device are projected into a single plane to integrate the spherical aberration spread over the *z* axis range. The bright region moves from left to right relative to the sperm head (arrows) (see Movie 3). Scale bars: 10 µm.

The human sperm head is elliptical with two flatter sides and narrower regions connecting them; in a way, it is like a flattened ellipsoid. When we observed the head rotating around its longitudinal axis, the narrow side behind the focal plane was clearly seen as a bright region, whereas the part of the head in front of the focal plane appeared as a dark region when the sperm head was inclined relative to the objective lens (Fig. 5A, images 4–6, see arrows). The contrast switch seen inside the sperm head was due to the head inclination relative to the objective lens (Fig. 5A, images 4–6): when the sperm head was parallel to the objective lens, no switch in contrast was seen (Fig. 5A, images 8 and 9), as was also demonstrated in the simulations described above. The 2D image sequence in Fig. 5A (images 4–7) shows that the bright region moved from left-to-right relative to the sperm head orientation, which is consistent with changes in inclination of the sperm head caused by head rotation. We found that this switch in contrast when the head is inclined is barely perceptible when observed with 2D microscopy (Fig. 2), as the sperm head continuously changes its position relative to the focal plane when swimming freely in the fluid (see Movie 1). A minimal focal change at the micron scale was found to be sufficient to prevent the detection of bright and dark regions induced by the sperm head inclination, as shown in Fig. 4C. Indeed, Figs 2 and 5A show that the inversion in contrast due to inclination of the sperm head was clearly visible for only a few focal planes as the piezoelectric device raised the objective. It would be a challenging task to change a single focal plane dynamically, at the microscale, to keep the sperm head exactly in focus to enable the detection of this change in contrast optical effect across the head using 2D microscopy, as shown in Fig. 4D. Our multi-plane system bypasses this challenge and allows unique detection of the accumulated changes in contrast from bright to dark regions induced by the sperm head inclination from a stack of multiple focal planes (Fig. 3), as detailed below.

### Detection of sperm head rotation direction from multi-plane image stacks

Taking advantage of the volume stack data we acquired, we summed the contrast switch caused by the head inclination occurring at different focal planes by integrating this multi-plane information in a single image. This circumvents the fact that the contrast switch across the sperm head was only observable for a few focal planes (as reported above). To this purpose, we calculated a 2D maximum intensity projection (MIP) image (Schindelin et al., 2012) for each rising slope *z*-stack containing 50 focal planes through the whole acquisition time of 3.4 s. The 2D MIPs of the *z*-stacks accumulated in a single image the maximum values of all the focal planes; for example, the bright regions in the images shown for the 2D image sequence in Fig. 5A. The integrated bright region induced by the switch in contrast when the sperm head was inclined manifested as a superimposed one-sided halo that was only present when the sperm head was inclined relative to the objective lens (Fig. 5B, images 4–7). Fig. 5B shows that the accumulated bright region moved from left to right relative to the head as time progressed (see images 4–7; Movie 3). The observed motion of the accumulated MIP bright feature over the course of time followed the direction of head rotation, as demonstrated in previous sections.

The directional motion of the superimposed bright regions was extracted from variations in the intensity profile along a segment across the sperm head (*bb′*, see red line shown in Fig. S1A). We employed different steps (see Materials and Methods section ‘Image processing steps for detection of sperm head rotation direction from multi-plane stacks) to detect the direction in which the one-sided bright halo moved with respect to the longitudinal axis of the sperm head (at a position placed at the front of the head, viewing the sperm from the tip of the head to the flagellum; Fig. S1).

### Validation of the detection of sperm head rotation using contrast switching of the sperm head

**Fig. 6.**
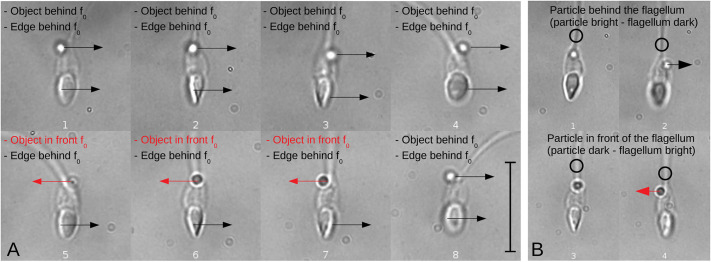
**Tracking a translucent particle attached to the neck of a spermatozoon, presenting the contrast inversion optical effect during a 360° turn of a free-swimming sperm.** (A,B) Numbered sequences of 2D single-plane images showing a spermatozoon with a particle of cell debris that became to the neck during free swimming in non-capacitating and low viscosity conditions. (A) Images showing a focal plane between the particle and the bright border of the spermatozoon head. Arrows indicate the directions of movement for the particle (object) and the bright border (edge). When the particle is above the neck of the spermatozoon (behind the focal plane) it appears bright, whereas it appears dark when under the neck (in front of the focal plane); simultaneously, the border of the spermatozoon is always behind the focal plane (and thus has a bright appearance), showing a CCW direction of rotation when viewed from head to tail. Note that the direction of the particle changes when it is dark (red arrows), as the particle rigidly follows the head rotating movement. (B) Images showing a focal plane between the particle and the flagellum. When the focal plane is placed between the particle and the flagellum, their relative position becomes evident: in images 1 and 2 (top), the particle (bright) is located behind the flagellum (dark) while moving to the right (black arrow), whereas in images 3 and 4, the particle is in front of the flagellum while moving to the left (red arrow). A CCW rotation is evident from these images. This shows that the described method is capable of detecting the direction of rotation. Scale bars: 10 µm.

The sperm head contrast inversion method to detect head rotation direction was validated by directly tracking the motion of a particle attached to the sperm neck during head rotation. Fig. 6 shows a spermatozoon with such a particle rigidly attached to its neck whilst rotating 360° during free-swimming motion. In the 2D single-plane image sequence shown in Fig. 6A, the focal plane was approximately positioned between the sperm and the particle along the *z* axis, in such a way that when the particle was behind the focal plane (Fig. 6A, images 1–4), it appeared to be bright, whereas when the particle was in front of the focal plane (Fig. 6A, images 5–8), it appeared to be dark. The arrow shown next to the particle indicates the direction of the displacement during head rotation, with a black arrow indicating that the particle was behind the focal plane (bright particle) and a red arrow indicating that the particle was in front of the focal plane (dark particle). As such, the particle could be observed to rotate in a CCW direction following the sperm head spinning motion: the particle moved from left to right when behind the focal plane (Fig. 6A, images 1–4) and moved from right to left when in front of the focal plane (Fig. 6A, images 5–8). On the other hand, the bright border arising from the contrast inversion when the sperm head was inclined relative to the objective lens always moved in the same direction relative to the sperm head orientation, from left to right, as indicated by the arrow shown on this bright region (Fig. 6A), regardless of whether the particle was in front of (Fig. 6A, images 5–8) or behind (Fig. 6A, images 1–4) the focal plane in each half cycle (see Movie 2). CCW rotation (as seen from head to tail) is expected to cause the bright region of the sperm head to always move from left to right relative to the sperm head, which is in agreement with the direction of particle rotation observed.

When the focal plane was placed between the particle and the flagellum (Fig. 6B), their relative positions became evident: while moving to the right, the particle (bright) was located behind the flagellum (dark) (Fig. 6B, images 1 and 2), and while moving to the left, the particle (dark) was located in front of the flagellum (bright) (Fig. 6B, images 3 and 4). The movement direction of the particle, combined with its relative position to the flagellum, allowed us to confidently conclude that rotation was in a CCW direction.

### Human sperm rotate with a conserved counterclockwise direction

We acquired images of 409 human spermatozoa that were freely swimming in 3D space: 180 under non-capacitating conditions and 229 in capacitating conditions. Both groups were studied in low- and high-viscosity media (see Materials and Methods). To avoid any modification to the contrast inversion optical effect used to determine the direction of head rotation, none of the images analysed in this work were pre-processed or enhanced. We found that in low-viscosity media, regardless of whether non-capacitating or capacitating medium was used, all the sperm spun in the CCW direction (as viewed from head to tail) when swimming freely. In contrast, in viscous and non-capacitating medium, 36% of the analysed sperm did not rotate at all (see Movies 4 and 5), whereas 64% spun in the CCW direction. Notably, in capacitating high-viscosity conditions these percentages changed: 18% of the analysed sperm did not rotate at all, whereas 82% spun with the conserved CCW direction. The results summarised in Table 1 suggest that capacitating medium also influences the ability of sperm to rotate in a viscous fluid. All the human sperm that we observed to rotate did so by turning in the CCW direction when seen from head to tail, regardless of the experimental condition.

**Table 1.**
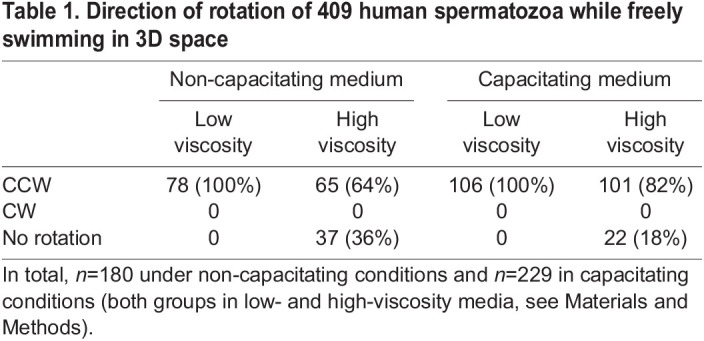
Direction of rotation of 409 human spermatozoa while freely swimming in 3D space

## DISCUSSION

Spermatozoa exhibit intricate combined translational and rotational movements, making their resulting motion in the laboratory frame of reference complex. When observed directly through a microscope in the laboratory frame of reference, these movements appear mixed, and they obscure the true motion relative to the moving frame of reference, translating and rotating with the sperm head (Gadêlha et al., 2020; 2021). To gain a comprehensive understanding of the true 3D motion of these cells, it is necessary to scrutinise both translational and rotational motions that spermatozoa perform during free swimming in 3D space (Ren and Bloomfield-Gadêlha, 2023 preprint). To investigate the genuine 3D movement of the spermatozoon flagellum (which involves the rotational components), a fixed-head frame of reference becomes indispensable. This frame is essential for inferring the complex relative intracellular movements, particularly between the head and flagellum, making it a critical aspect of analysis (Ren and Bloomfield-Gadêlha, 2023 preprint; Leung et al., 2021). For this, the rotation direction of the sperm head is fundamental.

Moreover, sperm flagellum elastohydrodynamics, mathematical modelling and image analysis have indicated that sperm head movement is highly dependent on forces imposed by the beating flagellum and its interactions with the local environment (Brokaw, 1982; Gadêlha et al., 2010, Gadêlha and Gaffney, 2019; Smith et al., 2009b; Ishimoto et al., 2017; Denissenko et al., 2012; Gaffney et al., 2011; Schatten, 1982; Lindemann and Lesich, 2016). Indeed, self-organised flagellar control models have demonstrated that mechanical attachment of the head can even dictate the travelling wave direction along the flagellum (Riedel-Kruse et al., 2007; Camelet and Jülicher, 2000; Oriola et al., 2017; Sartori et al., 2016; Lindemann and Lesich, 2021). The axoneme of mammalian sperm maintains a fixed position relative to the head, impeding their independent movement, and dynein interactions between the outer microtubule doublets exhibit a consistent chirality (Lindemann and Lesich, 2016, 2021). As a consequence, the forces responsible for propelling flagellar beating might operate in a specific chiral direction. The rotation of the head is a direct outcome of the helical component of the molecular action that powers the flagellar wave. When the beat becomes more flattened, such as in high-viscosity conditions before capacitation or in the presence of certain abnormalities, the rotation of the head decreases correspondingly, and progressive movement diminishes. Therefore, head/sperm rotation is thus necessary for helical swimming trajectories (Ren and Bloomfield-Gadêlha, 2023 preprint). These matters highlight the critical importance of directly establishing the head movement and its rotations in 3D space.

Despite the importance of head rotation for flagellar beat characteristics, as well as recent advances in high-speed 3D imaging and microscopy, to date there has been no agreement about the direction of rotation (see above; Muschol et al., 2018). Regardless of this context, direct methods to determine sperm head rotation direction are still lacking. Phillips has suggested that the rotation direction of mammalian sperm could be easily detected using 2D bright-field microscopy, by exploiting the fact that the sperm head is like a flattened ellipsoid and thus produces blinking ‘flashes of light’ as the head rotates around its swimming axis (Phillips, 1983; 1997). As the sperm head rotates, it displays a ‘flash of light’ travelling from left to right when looking at sperm from head to tail, similar to the sperm head orientation depicted in Fig. 5, and thus CW head rotation has been inferred. This, however, assumes that the ‘flash of light’ moves in the same direction as the sperm head rotation. We have demonstrated here that this assumption is inconsistent with the results of direct detection of head rotation direction (see Fig. 6). Instead, we have found that the true head rotation direction is opposite to the observed left-to-right movement of this ‘flash of light’. We have shown that this mismatch of movement between optical brightness and rotation of the object is accounted for by spherical aberration effects of the lens. Furthermore, translucent objects, such as the sperm head, are prone to perception bistabilities (Liu et al., 2012), which obscure the true direction of head rotation when analysing data from 2D microscopy, in addition to other unknown image inversions within microscopy systems (see Materials and Methods). These uncertainties, together with the combined use of 2D views of the sperm head trajectory to derive flagellar beat information, might have significantly contributed to the contradictory observations that are documented in the literature regarding the direction of sperm head rotation.

In the present study, we evaluated the head rotation direction of more than 400 spermatozoa across four conditions, namely non-capacitating and capacitating solutions of low and high viscosity. The high-viscosity condition was chosen to emulate the female cervical mucus (see Materials and Methods; Gaffney et al., 2011; Suarez, 2016). All rotating spermatozoa, regardless of the experimental condition, spun in a CCW direction as seen from head to tail.

It is important to mention that the rotation direction of each single spermatozoon was evaluated for a mean time of 3.4 s. A longer temporal analysis would be desirable in order to evaluate whether rotation direction changes over periods longer than 3 s, although unfortunately this is not possible with our present high-resolution 3D+t setup. Taken together, the summed analysed time of the 409 free-swimming sperm totalled 23 min, with no directional change observed. All analysed spermatozoa were at least 8 µm from the chamber bottom, and many were far above the coverslip (10–100 µm range), therefore minimal lower inner wall effects need to be considered (Smith et al., 2009b). Furthermore, the head rotation direction was the same for all cells analysed within the range mentioned.

Mammalian spermatozoa navigating in the female reproductive tract respond to wall architecture, fluid composition, temperature and flow. Sperm hyperactivation, an increase in sperm flagellar beating asymmetry occurring in the female tract, is part of sperm cell capacitation, preparing it for fertilisation (Suarez, 2016). Miller et al. (2018) have suggested that the asymmetric distribution of certain elements in the human sperm flagellar nanodomains (i.e. Hv voltage-gated proton channels) provides a structural basis for the selective activation of CatSper cation channels and subsequent flagellar rotation. The latter, together with hyperactivated motility, enhances sperm fertility.

Recently it has been shown that hyperactivation of bovine spermatozoa modulates sperm–sidewall interactions and, thus, navigation via female tract wall architecture. Specifically, hyperactivation has been observed to reduce the tendency of sperm to remain swimming along walls and to promote a response resembling chemotaxis (Zaferani et al., 2021b). More recently, Zaferani and Abbaspourrad (2023), have shown that flagellar beating propels sperm forward, and that the rolling component serves to counterbalance the effects of intrinsic asymmetry, maintaining the straight-line trajectory of the spermatozoa. Nevertheless, when sperm enter a viscoelastic fluid, this rolling component diminishes, leading to a reversible transition from progressive planar motion to chiral motion. This transition highlights that swimming without rotation remains possible, albeit through a change to a less progressive mode. In this context, it is worth highlighting our finding that human spermatozoa in low-viscosity media, independently of their capacitation state, all rotate in a CCW direction. In contrast, in high-viscosity conditions, 36% of spermatozoa in non-capacitating medium were found to not rotate at all, with most of them (82%) undergoing CCW rotation when incubated in capacitating medium. This notable change in head rotation under capacitating conditions in high-viscosity conditions, as well as how sperm head rotation and flagellar rolling influence swimming trajectories in 3D space and during fertilisation, needs to be fully explored, as it might unveil an aspect of the essential need for capacitation in fertilisation.

In this work we have shown that 2D microscopy alone cannot distinguish the rotation direction of axially symmetric, streamlined, translucent, swimming human spermatozoa. Methods employing such imaging techniques might need reassessment, and with the exception of the present 3D+t-based work, no methodology is currently available to directly measure the head rotation direction of human spermatozoa. We have demonstrated that the human sperm head rotates with a robust, conserved and recoverable CCW direction of rotation, as viewed from head to tail. Our observations reconcile structural information of mammalian sperm flagellar architecture, including conserved chirality in the axonemal driving unit, with a conserved direction of rotation for human spermatozoa, regardless of viscosity and capacitating conditions. The ability of human spermatozoa to fertilise might be intimately related to head rotation, as in the present work capacitating medium was observed to excite a larger proportion of the population to rotate in high-viscosity conditions. Finally, the methods described here can be applied to other free-spinning objects and rotating microorganisms that possess an axially symmetric body architecture similar to that of human spermatozoa.

## MATERIALS AND METHODS

### Ethical approval for human semen samples

The bioethics committee of the Institute of Biotechnology, Universidad Nacional Autónoma de México approved the protocols for the handling of human semen samples, and the study was conducted according to the principles expressed in the Declaration of Helsinki. Donors were properly informed regarding the experiments to be performed, and each donor signed and agreed to a consent form. All samples fulfilled World Health Organisation of 2021 requirements for normal fertile semen samples.

### Culture media

Human tubal fluid (HTF) medium was used in this study as the low-viscosity medium. Non-capacitating HTF medium (pH 7.4) contained 4.7 mM KCl, 0.3 mM KH_2_PO_4_, 90.7 mM NaCl, 1.2 mM MgSO_4_, 2.8 mM glucose, 1.6 mM CaCl_2_, 3.4 mM sodium pyruvate, 23.8 mM HEPES and either 21.4 mM or 60 mM sodium lactate (because papers report the use of both these conditions). Capacitating medium (pH 7.4) was HTF medium supplemented with 5 mg/ml BSA and 25 mM NaHCO_3_. Capacitating medium (pH 7.4) used for recording was only supplemented with 25 mM NaHCO_3_; BSA was not added. No significant differences were found in the sperm head rotation characteristics regarding the lactate content of HTF medium (data not shown). The high-viscosity medium was HTF medium containing 1% methyl cellulose [Sigma-Aldrich, M0512; viscosity of 4000 cP (4 Pa·s) for a 2% solution in water], which was estimated to have a viscosity of 0.14 Pa·s at 37°C. This viscosity was selected as it is close to the one present in the fluid of the female reproductive tract (Gaffney et al., 2011; Suarez, 2016). Methyl cellulose (1%) was added to non-capacitating or capacitating HTF recording medium, depending on the experiment.

### Biological preparations

Semen samples were obtained by masturbation from healthy donors after 48 h of sexual abstinence. Highly motile sperm were recovered after the following swim-up protocol. Briefly, 300 µl of semen was placed in a test tube, then 1 ml of non-capacitating or capacitating medium, depending on the experimental condition, was added on top. Tubes were incubated at a 45° angle, at 37°C in a humidified atmosphere of 5% CO_2_ and 95% air, for 1 h. Then, sperm from the medium on top were collected, and the concentration was adjusted to 10^6^ cells/ml. To promote *in vitro* capacitation, sperm in capacitating medium were incubated for an additional 5 h. Recordings were performed in non-capacitating low- and high-viscosity media, and in capacitating low- and high-viscosity media.

### Sperm samples

A total of 409 freely swimming spermatozoa (across 30 samples, one per day, from nine different donors) were analysed: 180 in non-capacitating media (78 in low-viscosity medium and 102 in high-viscosity medium) and 229 in capacitating media (106 in low-viscosity medium and 123 in high-viscosity medium).

### 3D imaging microscopy

Image stacks comprising multiple focal planes were acquired with the system originally described by Corkidi et al. (2008), which consisted of an inverted Olympus IX71 microscope mounted on an optical table TMC (GMP SA, Switzerland), reconfigured with a piezoelectric device P-725 (Physik Instrumente, MA, USA) that periodically displaced a high-magnification 100× objective (Olympus UPlanSApo 100×/1.4 NA oil objective) at a frequency of 80 Hz with a *z* displacement of 20 µm. Spermatozoa swam in a custom-made open imaging chamber (no glass above) for the inverted microscope. Dimensions were 20 mm×3.5 mm (diameter×depth), for a total volume of ∼1 ml. A coverslip (VWR, 48380-080) sealed the bottom of the chamber, which was filled to its full capacity. The cells were recorded around the centre of the chamber to avoid interactions with the vertical wall. Experiments were conducted in two spatial regions, at least 8 µm from the chamber bottom and far above the coverslip (range 10–100 µm). A high-speed camera NAC Q1v (Nac Americas, Inc., USA) acquired 8000 images/s with 640×480 pixel dimensions. This high-speed acquisition, which was necessary to obtain sufficient sperm head rotation focal planes, limits spatial resolution. Every rising movement of the piezoelectric device (half cycle, i.e. 1/160 s) obtained 50 different focal planes (one image per focal plane). The high-speed camera could acquire up to 28,000 images (using 8 GB RAM), thus could record rotating head motion for a total of 3.4 s. To confidently determine sperm head rotation direction, the objective of this work, every possible inversion in each single element in the path of the optical–electronic pipeline had to be carefully considered [the inverted microscope, camera driver setup and image-processing software including that used for visualisation (Fiji, Matlab and Paraview)]. As a control test, we placed in the microscope stage (using a 4× objective) a known pattern (an R character on a piece of paper) facing the objective (upside down if seen from the top of the microscope). We verified that the character appeared upright in the computer screen and that it moved accordingly with horizontal and vertical stage movements, when observing the stage from the bottom to top direction, where the objective was placed.

### Numerical simulations of weak phase objects

The simulations of the imaging process presented in Fig. 4 were calculated and graphed using MatLab. The matrices used in the simulation had a total size of 128×128 pixels with the unrotated object occupying the central 40×40 pixels. No scattering from the object was considered, and the phase introduced by the object was set to 0.1 radians, which is a typical value for weak phase objects. This object was geometrically rotated to the required position for each case and the defocus and spherical aberration were introduced to the diffraction equation using equations (1) and (2). The contrast inversion effects were observed in the calculated and graphed diffraction patterns.

### Image processing steps for detection of sperm head rotation direction from multi-plane stacks

#### Tracking sperm head position

To track the sperm head position, we used the method described by Corkidi et al. (2021). This method provides the dominant orientation of spermatozoa over time (angle φ in Fig. S1A) relative to the microscope fixed frame of reference, depicted by the line *C*. The centre of the sperm head over line *C* is defined as *a*. These two experimental parameters define the position and orientation of the major axis of the sperm head represented by *C* (see Corkidi et al., 2021). By construction, *C′* is perpendicular to *C*, and the intersection point is at *d* µm from *a* (a third of the average size of the long axis of the human sperm head). Finally, *b* and *b′* are located symmetrically along *C′* at a distance *b* from the intersection of *CC′*. The pixels along *bb′* (represented by the red line in Fig. S1A) were used to measure the intensity profile over the 2D MIP image (see Movie 6). The direction of motion of the maximum intensity of the profile series [by using variation in position of the weighted average (see next section) along the segment *bb′,* as shown in Fig. S1C,D] defines the sperm head rotation direction. The equation describing the points along the line *C* is *y*=(*tanφ*)*x*+(*y*_*a*_−*x*_*a*_*tanφ*), the intersection point between *C* and *C′* is given by the condition 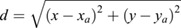 and its coordinates (*x*_*d*_, *y*_*d*_). The equation describing the points along *C′* is defined by 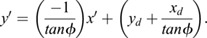 The points *b* and *b′* are positioned at distances 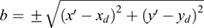 along *C′* centred at the intersection with *C*. The set of points that are embedded in the *bb′* segment is 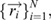 where *N* is the number of points belonging to *bb′* (red line in Fig. S1A). From this, the grey level profile over the segment ***bb****′* can be quantified.

#### Determination of the sperm head rotation direction

The position of the weighted average of each intensity profile along *bb′**,*** while the head is turning, is quantified to establish the head rotation direction. The weighted average position over *bb′* is 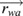, analogous to the ‘centre of mass’ of the intensity levels along *bb′*. Tracking 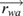 over time reveals the movement of the brightest part of the sperm head in the 2D MIPs. This feature applied to the grey level profile over the line segment *bb′* (see Fig. S1) is expressed in Eqn 4:
(4)

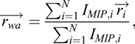
where *I*_*MIP*,*i*_ is the grey level value of the corresponding 2D MIP image position 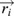. If 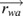 moves from *b* to *b′*, the cell is rotating CCW, and if 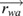 moves from *b′* to *b*, the cell is rotating CW, as shown in Fig. 5 and Fig. S1.

Fig. S1A and B show the segment *bb′* positioned over the sperm head in two consecutive timepoints, with the bright border moving from *b* to *b′*. The corresponding grey levels of the 2D MIP profiles are plotted beneath each image in Fig. S1C and D. Fig. S1E shows the values of the weighted average for two and a half head turns, as shown in Movies 3 and 6. Red circles over the first minimum and maximum in Fig. S1E correspond to the first half turn. Minima denote that the weighted average of the intensity profile is shifted towards the *b* side of the sperm head [taking *b* (from *bb′*) as the origin for the profile], whereas maxima denote a shift towards the opposite *b′* side. Fig. S1F shows the corresponding intensity profile (*bb′*) kymograph: the lower dashed lines point out the bright profiles corresponding to Fig. S1A and B, denoting a 180° head turn. The upper time-ascending arrow from left to right of the kymograph denotes a CCW head turn.

### Invariance of the method to sperm orientation

To investigate whether the direction of sperm rotation could be an artifact of the illumination setup of the microscope (i.e. whether the bright border of the sperm head edge was an effect of the illumination setup), we analysed the rotation of non-capacitated sperm swimming in four different directions in a Cartesian plane. As we explained above, in the sequence shown in Fig. 6, it can be seen clearly that the border of the narrowest part of the head is naturally marked with a bright semi-circle. This bright feature turns in the direction of the sperm head (note that this bright border is located behind the sperm head, as explained above). We verified that, independently of the swimming trajectory of the sperm, the evolution over time of this bright semi-circle clearly defined the direction of rotation of the sperm head. Fig. S2 shows two consecutive timepoints of four different sperm with outgoing trajectories from the centre of each of four Cartesian planes. As can be seen in Fig. S2, the bright region (which occurs due to the optical spherical aberration contrast inversion effect) in each sperm always appears first on the *b* side of the head and then moves to the opposite *b′* side at the subsequent timepoint (see Fig. S1). This indicates that all the sperm rotate in a CCW (since the bright border is behind the head of the sperm) direction independently of the direction of the sperm trajectory (four quadrants).

## Supplementary Material

10.1242/joces.261306_sup1 Supplementary information
