# Supplementary material for: Human sperm rotate with a conserved direction during free swimming in four dimensions
Source: J Cell Sci. 2023 Nov 29;136(22):jcs261306. doi: 10.1242/jcs.261306 (PMC10729817; doi:10.1242/jcs.261306)
Supplement: Supplementary information [file joces-136-261306-s1.pdf]

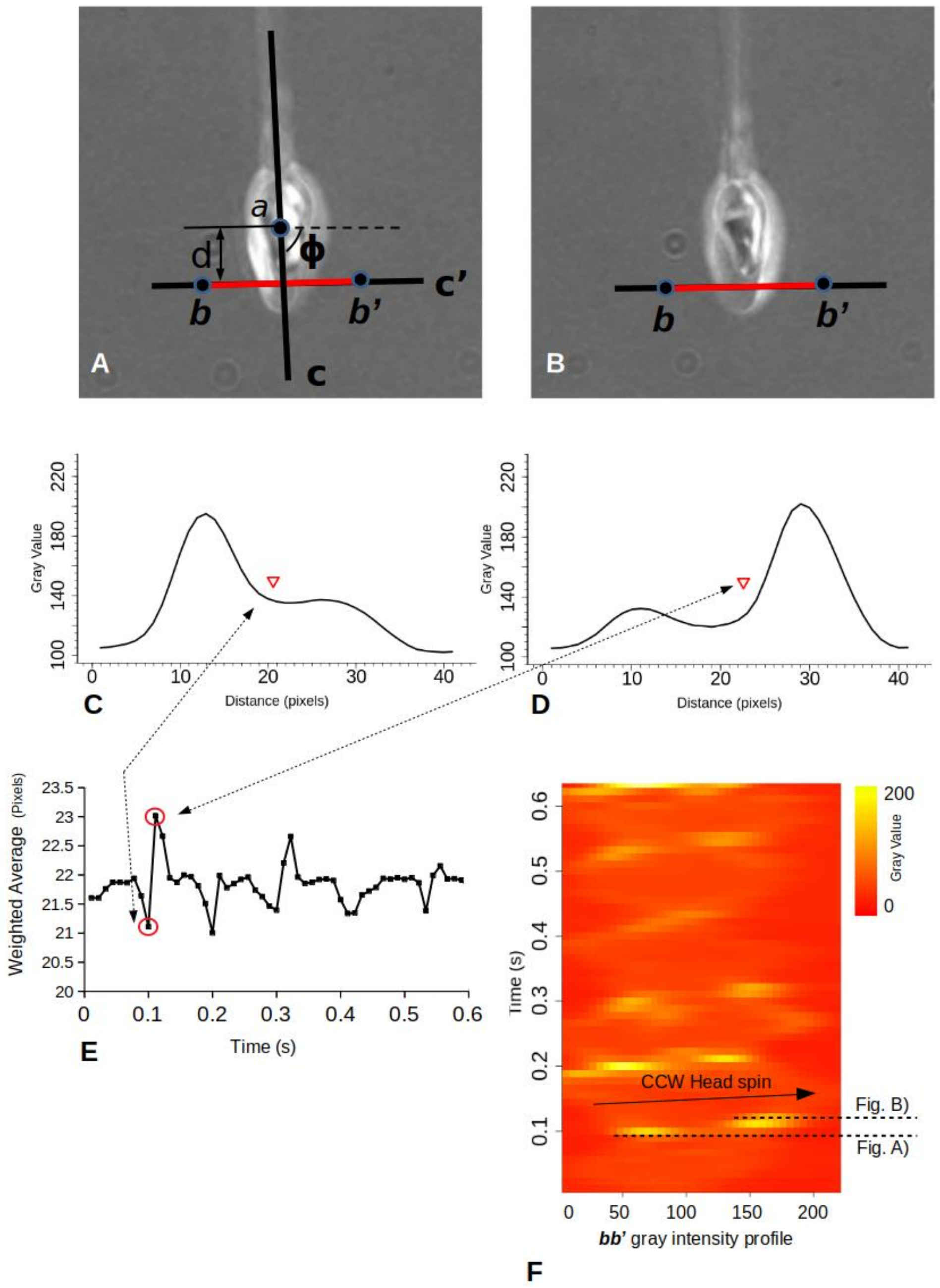

Fig. S1. Sperm head bright region travels with a conserved direction. (A-B) The tracking process estimates the angle ϕ of the dominant direction of the spermatozoon relative to the microscope frame of reference and defines the orientation of the sperm (line **C**, see Corkidi et al., 2021). The straight-line **C'** is perpendicular to **C**, and the intersection point is at d μm from head centre (1/3 the average size of the long axis of the human sperm head). ***b*** and ***b'*** are located symmetrically along **C'** at a distance ***b*** from the intersection of **CC'**.

Journal of Cell Science • Supplementary information

The coordinates of ***bb'*** (represented by the red line) are used to measure the intensity profile over the 2D MIP image (see Video S1). (C-D) The corresponding gray level of the 2D MIP profiles (from A-B) with its centre of mass depicted by the red triangle. (E) Weighted average of 2D MIP profiles for two and a half head turns. Minima denote that the weighted average of the intensity profile is shifted towards the ***b*** side of the sperm head (taking ***b*** (from ***bb'***) as the origin for the profile) while maxima denote the shift towards the opposite ***b'*** side. The transition from a minimum to a maximum corresponds to half a turn of the sperm head in a CCW direction (two and a half CCW turns for this series). (F) Intensity profiles (***bb'***) kimograph; dashed lines point out the bright profiles corresponding to Figures A and B denoting a 180 degrees head turn. The lower time ascending arrows show waves of the brightness level travelling from left-to-right, indicating CCW head rotating direction when seen from head to tail.

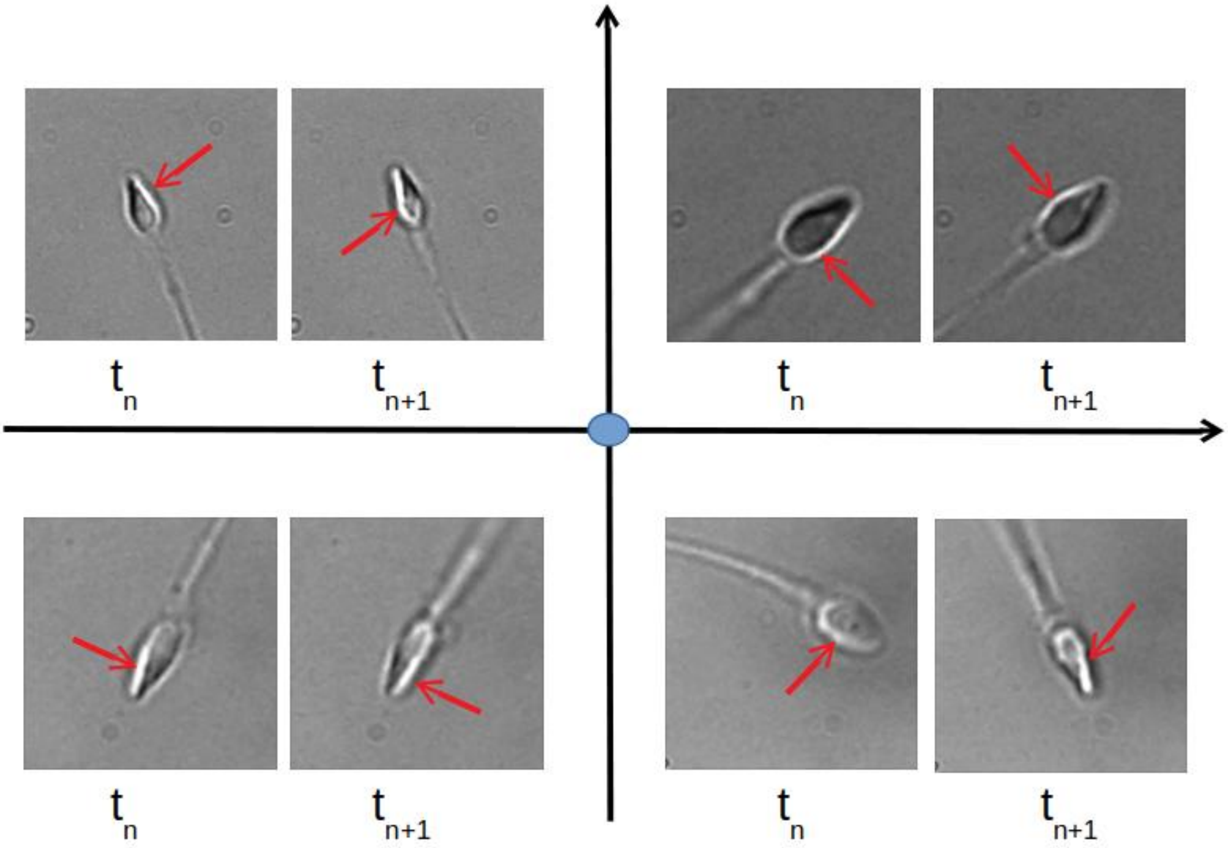

Fig. S2. Four different non-capacitated sperm with outgoing trajectories from the centre of each of the four Cartesian planes (blue circle). Two different time-points (t_n and t_{n+1}) are shown for each sperm. The bright region (due to the optical spherical aberration contrast inversion effect) always appears first in the left side of the head and then moves to the right side at the subsequent time-point (from head to tail) indicating a CCW head rotating direction as shown in Results. The red arrows indicate the bright border in each image.

Journal of Cell Science • Supplementary information

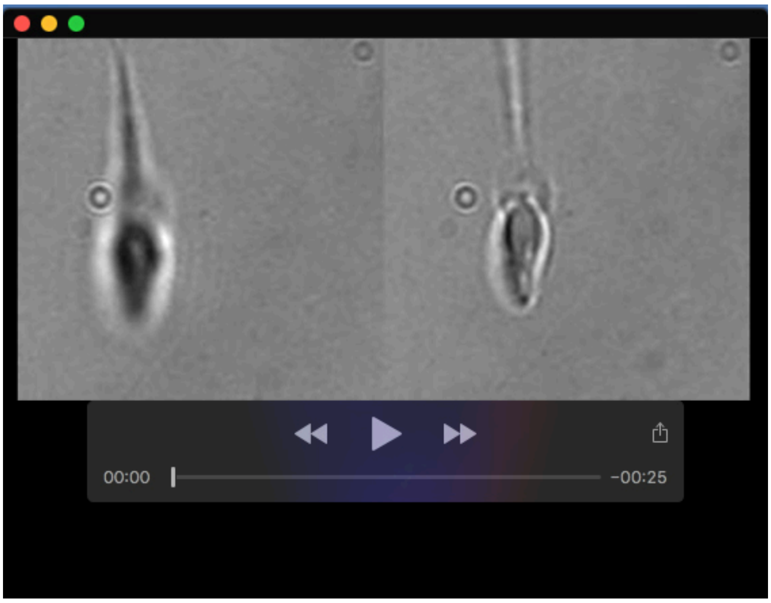

Movie 1. This video shows two views of the same spermatozoon at two different focal planes separated by 2 µm. In the left panel, the determination of the head rotating direction is not possible, while in the right panel, the bright flash moving from left-to-right gives the information to infer the rotating direction of the cell (note that this bright flash is located behind the translucent head, indicating a CCW rotating direction, seen from head-to-tail).

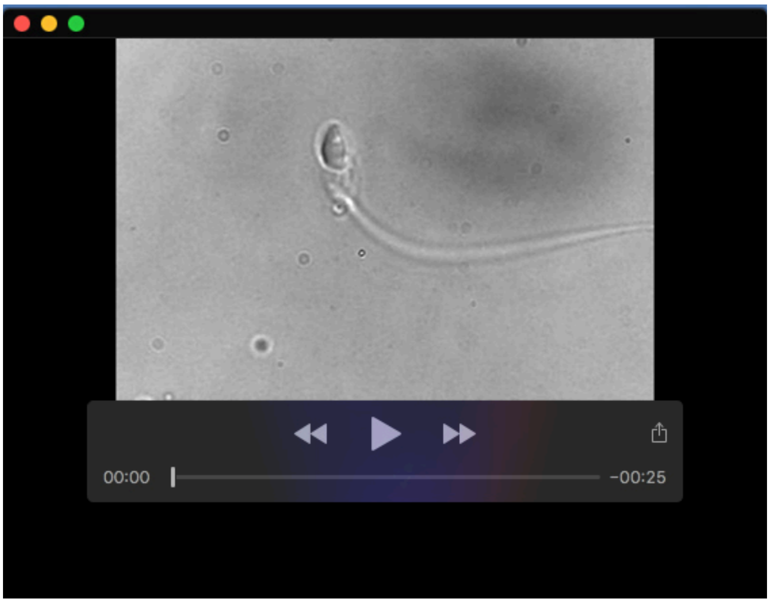

Movie 2. A sperm cell with a translucid particle rigidly attached to its neck, whilst rotating 360 degrees during free-swimming motion.

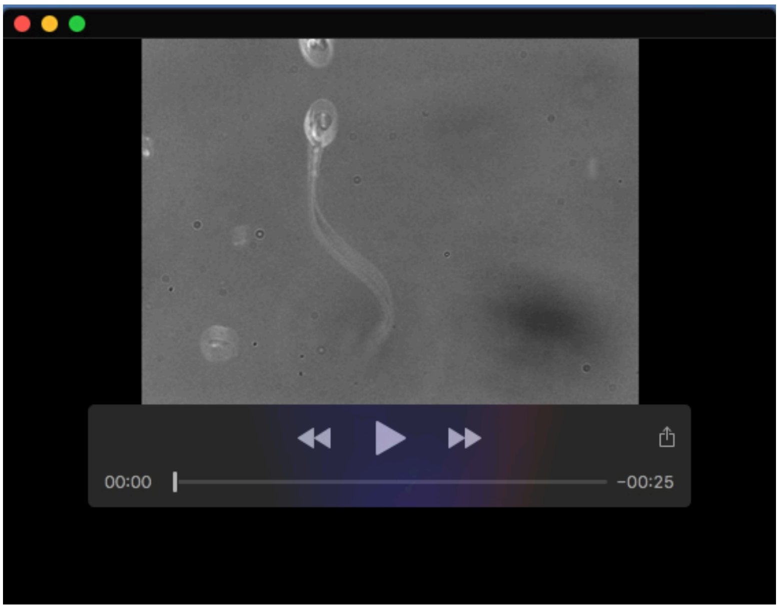

Movie 3. The 2D MIP of the z-stack accumulates in a single image the maximum values of all the focal planes, i.e. bright regions for a time sequence. A bright region moves from right-to-left relative to the head as time progresses. At the final part of the video, another spermatozoon appears swimming in the opposite direction, showing the bright region to move consistently in the inverse direction (see also Supporting Information and Fig. S2).

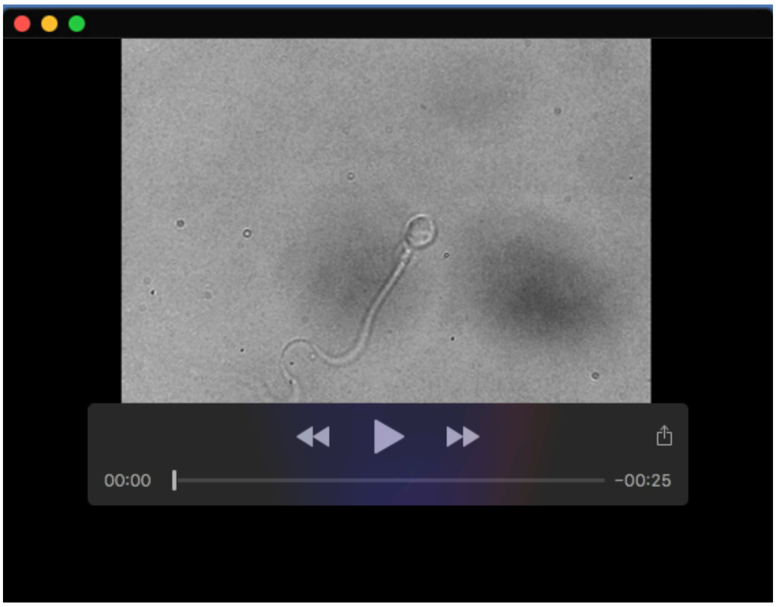

Movie 4. Spermatozoon swimming in non-capacitating viscous medium with absence of head rotation.

Journal of Cell Science • Supplementary information

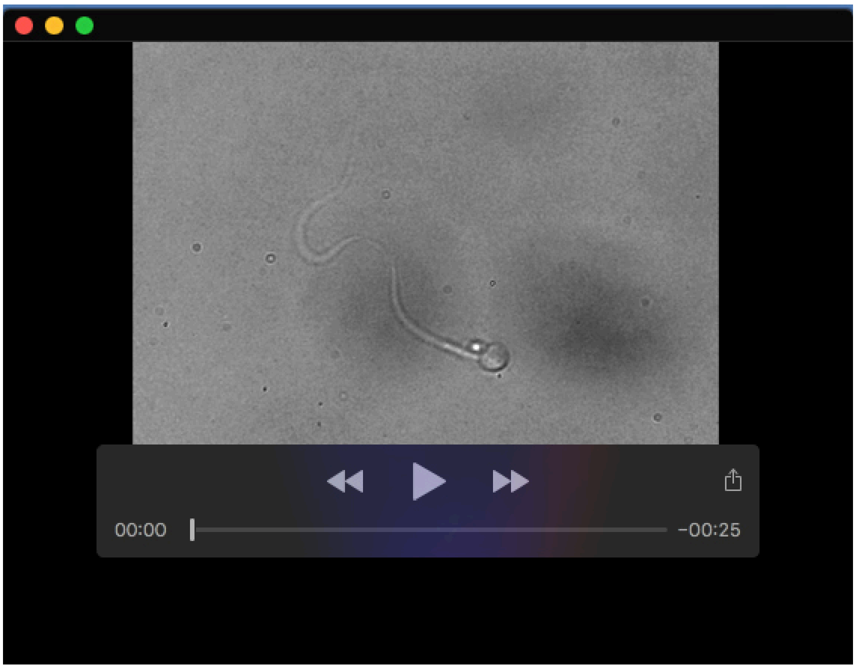

Movie 5. Spermatozoon with translucent particle stuck on the neck, swimming in non-capacitating viscous medium with absence of head rotation.

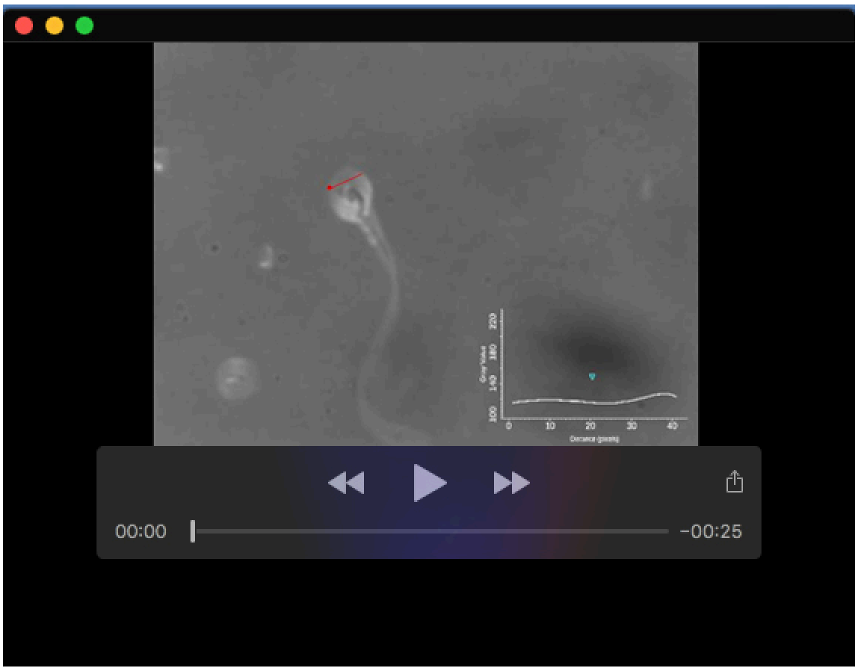

Movie 6. Gray level of the 2D MIP profiles (over the tracked red line ***bb'*** from Fig. S1) with its centre of mass depicted in the graph by the blue triangle.
